# The Southern Surgical and Gynecological Association

**Published:** 1889-12

**Authors:** 


### THE SOUTHERN SURGICAL AND GYNECOLOGICAL ASSOCIATION.

In this number of the JOURNAL we present to our readers the proceedings of the annual meeting of the Southern Surgical and Gynecological Association, which was held at Nashville last month. We feel that this is an Association in which every Southern physician may feel a pardonable pride. The work accomplished and the papers presented at the last meeting present a gratifying evidence of the progressive spirit which animates the profession of the South in the two specialties to which this society is devoted. Much of the work presented was original in character and eminently practical in its nature. The Association will not suffer by comparison of its work with that of any other special society in this country.

When it is remembered that this Association sprung into existence only a year ago, the success which it has achieved is truly wonderful. The Nashville meeting attracted visitors from the largest medical centers of the North, who added largely to the interest of the occasion, by their able papers and discussions. The bulk of the work however, was in the hands of Southern surgeons and gynecologists, and will contribute to the elevation of the profession at home and the establishment of its reputation abroad.

We are gratified to learn that the next meeting of the Association will be held in Atlanta, and we bespeak for it a hearty welcome and a cordial hospitality.

